# The architecture of the primary mental healthcare system for older people in India: what public policies tell us

**DOI:** 10.1186/s13033-021-00494-8

**Published:** 2021-08-30

**Authors:** Tom Kafczyk, Kerstin Hämel

**Affiliations:** 1 Department of Health Services Research and Nursing Science, School of Public Health, Bielefeld University, Universitaetsstrasse 25, 33651 Bielefeld, Germany; 2 Department of Health Services Research and Nursing Science, School of Public Health, Bielefeld University, Universitätsstrasse 25, 33615 Bielefeld, Germany

**Keywords:** Mental health services, Primary healthcare, India, Health services for the aged, Alternative therapies, Policy analysis, Informal care, Community health workers

## Abstract

**Background:**

Old age mental healthcare is an issue that cuts across old age, general health, and mental healthcare policies. While strengthening the primary mental healthcare system in India is a common strategy across policy fields to improve the mental health of older persons, very little is known about the system’s planned architecture. This study explores public policy strategies for and approaches to the mental health of older persons, focusing on the primary healthcare (PHC) level and the role of the family.

**Methods:**

A document analysis of 39 key public national policy documents (2007 – 2019) from three thematically grouped policy fields (mental health, old age, and general health) was conducted.

**Results:**

Comprehensive community-based primary mental healthcare – focusing on vulnerable population groups including older persons – has been strengthened significantly since 2007. The promulgated approaches and strategies build on traditional community-based approaches to mental healthcare in India. They focus on (a) integrating community health workers into primary mental healthcare, (b) empowering the community to participate in healthcare planning, implementation, and monitoring, (c) supporting the family through a family-led approach to mental healthcare, and (d) integrating traditional Ayurveda, Yoga and Naturopathy, Unani, Siddha, Sowa-Rigpa and Homeopathy (AYUSH) services into primary mental healthcare.

**Conclusions:**

While all policy fields address mental PHC, they do so in different ways, and approaches and strategies that promote an integrated perspective across policy fields are lacking. To realize the policies vision, strengthening PHC will be essential. Further research should evaluate strategies and approaches in light of social developments, such as eroding family norms and the poor state of the public health system in India, in order to assess their opportunities, challenges, and overall feasibility, with the benefits older people would experience taking centre stage in these inquiries.

## Introduction and aim of the study

Community-based mental healthcare has a long tradition in India [1]. In 1982, the National Mental Health Programme (NMHP) strengthened the integration of mental healthcare at the primary healthcare (PHC) level [2–4]. This effort was reinforced by a strategic vision, the District Mental Health Programme (DMHP), which established the district as the territorial unit for mental health program implementation [5]. In the last decade, the government of India has further framed and developed the mental healthcare system through legislation, strategies, and programs (for a detailed analysis see Kafczyk and Hämel [6]). In this context, the need to develop mental health measures at the PHC level has been repeatedly addressed in policies [7]. However, gaps in policies for older persons persist [6]; furthermore, India does not yet have a national strategy on dementia [8].

Older people constitute one of the fastest growing population groups in India [9] that is vulnerable to impaired mental health, such as depression, anxiety or dementia [10, 11]. This public health problem is not exclusive to India; it is growing in importance in other low- and middle-income countries (LMICs) as well [12].

Older persons should particularly benefit from a PHC-oriented approach, which would enable them to address their mental health issues close to their homes [13, 14]; this holds particularly true for older persons who are dependent on support [15].

However, research on how policies in India envision a primary mental healthcare system capable of addressing the growing number of older people in need of mental healthcare is largely lacking.

Policy attempts to further develop primary mental healthcare structures for older people in India should be analysed against the background of poor access to primary mental health services for older people in India [14, 15]. For example, it is estimated that the prevalence of depression in old age is 34.4% in India [16], while only ~ 17.1% of affected older persons receive treatment [17]. One study by Patel and Prince [13] describes the primary care system in Goa as having low awareness of and poor preparedness for mental health problems in older age [15]. In rural areas, where the majority of older persons reside [18], the situation is generally worse [14, 19–22].

A proper analysis of mental healthcare policies must consider that the family is the most reliable caregiving resource for older people in India; this holds true for mental healthcare [15, 23–25]. Even stronger family involvement in mental healthcare for older family members has been called for to improve the situation [13, 20]. The realization of this call is challenged by changing family structures and norms in India [14, 15, 26–29]. Consequently, research should explore the extent to which policies anticipate these changes.

This study explores how national Indian policies frame primary mental healthcare for older persons in India. We focus on the following research questions:

1. What role has been assigned to the primary care level in providing mental healthcare for older persons?
2. Which approaches and strategies are envisioned to promote the mental health of older people and to provide services for those in need at the PHC level?
3. How are family caregivers addressed in these approaches and strategies?

Before examining these questions, we will first look more closely at the contours of the primary mental healthcare system in India, to which these policies seek to link.

### Context of the study: primary (mental) healthcare in India

India has over 1.3 billion inhabitants, and 8.6% of the population is aged 60 years and older [30]. A selection of India’s social, economic and health indicators is presented in Table 1. However, it must be noted that the country is characterized by strong regional disparities in terms of social and economic development as well as access to public services.

**Table. 1:** Selected demographic, economic and primary (mental) healthcare system indicators in India

Indicator	Data
Demographic and socioeconomic	
Total population (2018) [107]	1,352,642,000
Females as a % of the total population (2018) [107]	48
Total population living in rural areas in % (2011) [18]	69
Population 60 + years (millions of inhabitants in 2011) [18]	104
Females 60 + years in % (2011) [18]	52.2
Population 60 + years living in rural areas in % (2011) [18]	71
Population 60 + years expected in 2050 (in million) [108]	330
Life expectancy at birth for both sexes (2018) [107]	68
Effective literacy rate in % of the total population (2011) [18]	73
Effective literacy rate in % of the population 60 + (2011) [18]	44
Human Development Index (2019) [109]	0.645
Gini coefficient (2010–2018) [109]	37.8
Public health expenditure	
Gross domestic product (GDP, US$ per capita in 2019) [110]	2099.60
Domestic general government health expenditure (GGHE-D) as a percentage of general government expenditure (GGE) in % (2017) [107]	3.4
Domestic private expenditure on health as a % of current health expenditure (2018) [111]	72.4
Mental health expenditure as a % of the total health budget (2011) [46]^a^	0.06
Government health expenditure on primary care as a % of total health expenditure (2016–17) [112]	52.1
Characteristics of the health system	
Population with health coverage in % (here of % covered by public insurance; 2017–2018) [113]	37.2 (78)
PHC with gatekeeping function to specialized care [114]	Limited gate keeping function
Registration at a PHC centre	Yes
Predominant mode of provision in primary and specialized care	Private
Predominant organization in specialized ambulatory care	Hospital outpatient departments
Portfolio of services defined at the central level	Yes
Freedom of choice of doctors in primary care	Yes
Mental health facilities	
Mental health outpatient facilities (per 100,000, absolute number in parentheses, 2011) [46]	0.33 (4000)
Psychiatric beds in general hospitals (per 100,000, absolute number in parentheses, 2011) [46]	0.82 (10,000)
Mental hospitals (per 100,000, absolute number in parentheses, 2011) [46]	0.004 (43)
Beds in mental hospitals (per 100,000, absolute number in parentheses, 2011) [46]	1.47 (17,835)
(Mental) health resources	
Psychiatrists (per 100,000; 2011) [46]	0.3
Nurses (per 100,000; 2011) [46]	0.17
Psychologists (per 100,000; 2011) [46]	0.05
Social workers (per 100,000; 2011) [46]	0.03

^a ^Due to the federal system of governance in India, each of the 35 states and union territories/administrations has its own mental health budget; the number refers to the Central Government

**Fig. 1:**
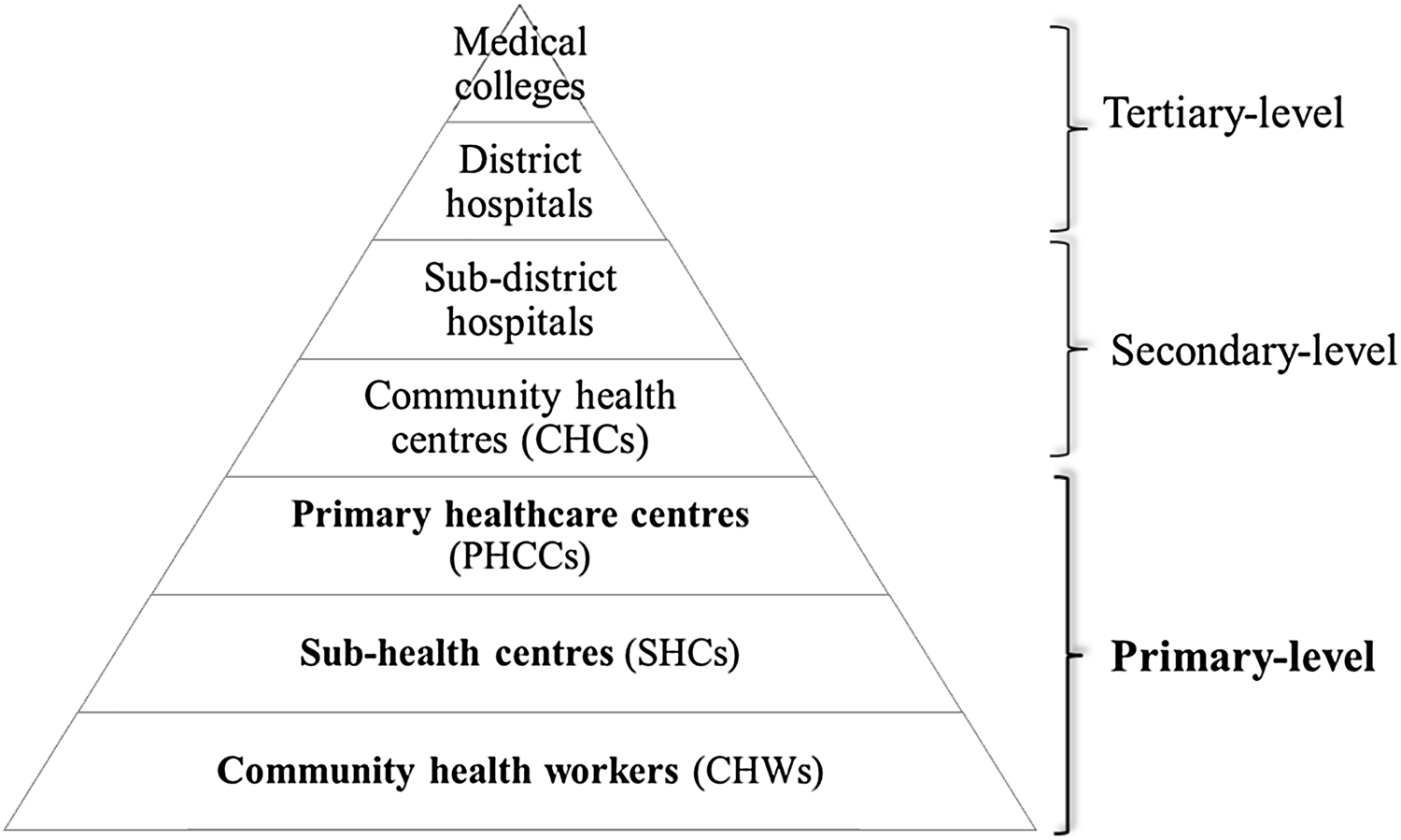
The health system structure in India. Own figure, based on Chokshi, Patil [32]

India adopted a three-tiered health system model [31, 32] following the recommendations of the Bhore Committee (1943). It is based on the idea of a strong PHC providing universal access to healthcare close to people’s homes in all regions (Fig. 1) [33].

Further key principles of the PHC architecture are multiprofessional provision of integrated, preventive, and curative health services, including mental healthcare, and community participation in healthcare delivery [33]. Rural development takes centre stage in Indian primary healthcare policies [34]. After the Bhore Committee’s recommendations, health planning envisaged the establishment of sub-health centres (SCHs) and primary healthcare centres (PHCCs) at the PHC level, supplemented by community health workers (CHWs) and semiprofessional health workers who live in the community they serve and who function as the link between India’s population and the healthcare system [31].

Since 1975, Anganwadi workers (AWWs) and auxiliary nurse-midwives (ANMs) have been the major CHWs in India; these providers focus on women and child health [35]. In addition, Accredited Social Health Activists (ASHAs) were introduced in 2005 by the National Rural Health Mission (NRHM) to improve the outreach and coverage of health services in rural areas. Each village in India is supposed to have at least one ASHA and one AWW [35]. ASHAs represent a new kind of CHW; they supplement the work of AWWs and ANMs by also addressing noncommunicable diseases (NCDs) [36]. Approximately 800,000 ASHAs are deployed in India today [35].

Interestingly, the basic elements of rural PHC have been increasingly adopted in urban regions. In 2011/12, against the background of rapid urbanization in India [37], the Government of India launched the National Urban Health Mission (NUHM) with the aim of reorienting the healthcare system in urban areas toward the model used in rural areas [38]. The NUHM and NRHM are now implemented as the National Health Mission (NHM). The main function of the NHM is to provide a vision for India’s health system and to consolidate different health programs at an operation level [39].

In 2018, the PHC infrastructure in India encompassed 158,417 SHCs and 25,743 PHCCs, each typically serving 5000 and 30,000 people, respectively [39]. The SHCs and PHCCs are public institutions run by the Ministry of Health and Family Welfare (MoH&FW) and the state governments [32]. SHCs are staffed by at least one ANM and one male health worker; they provide maternal and child health services, family welfare, nutrition, immunization, diarrhoea control and communicable disease care, and health education designed to bring about behavioural change [32]. They also increasingly provide non-communicable disease care [40]. PHCCs are patients’ first contact point with a medical officer and paramedical and other staff. These workers are tasked with providing integrated preventive and curative healthcare [32], including mental healthcare [41].

It must be noted that India is a country of medical pluralism, with traditional services being frequently utilized, particularly by persons with mental illnesses. Furthermore, faith-based rituals and procedures are common practice to ‘heal’ mental disorders [42]. For many older persons, traditional services are more accessible or acceptable [15]. Recognized by the Government of India as alternative systems of medicine are Ayurveda, Yoga and Naturopathy, Unani, Siddha, Sowa-Rigpa and Homeopathy (AYUSH); in 2018, there were 799,879 registered AYUSH practitioners in India [39]. With the introduction of the NRHM in 2005, initial attempts were made to integrate AYUSH into the public PHC system [42].

However, despite the need to improve primary mental healthcare in India, there are still few resources available [43]. Moreover, public investment in (primary) healthcare has not kept pace with the economic growth of India in recent years [44]. The training and supply of health professionals in the country has also not been increasing as expected [43]. While the median percentage of government health budget expenditure on mental health is nearly 2.0% globally [45], this rate is only 0.5% in low-income countries [46]; even worse is the situation in India, where mental health expenditure reaches only 0.06% [46]. In contrast to the delineated policies, mental healthcare in India concentrates on urban areas and is mainly provided in secondary and tertiary care facilities [15, 19, 27]. It is the private sector that has grown in importance and now plays an important role in the Indian mental health system [15, 27]. At present, access to and provision of primary mental healthcare are still poorly developed in rural areas and in particular the northern parts of India [15].

## Methods

This study explores how national Indian public policy documents from 2007 until 2019 frame the primary mental healthcare system for older persons in India. Our analysis is informed by the ‘policy triangle model’ developed by Walt and Gilson [47]. We conducted an in-depth analysis on the context, actors and processes and inclusion of older persons in (primary) mental healthcare beforehand [6]. In this analysis, we grouped the identified policies in the three distinctive policy fields: (a) mental health, (b) old age and (c) general health. We confirmed that primary mental healthcare for older people is a cross-cutting policy issue, as several federal-level ministries have authored relevant policies (see also Table 2) [6]. We have furthermore shown that policies were developed within the context of increased international attention to old-age mental healthcare and increased domestic recognition of population ageing and the decline of family care potential. Policies increasingly recognise older people as being vulnerable to poor mental health [6]. However, our analysis also revealed that the development of old-age-inclusive primary mental healthcare is still in its infancy in India with a need for unambiguous and integrated policy approaches.

**Table. 2:** Included national policies relevant for old-age mental healthcare

Policy field	Policy (included documents)	First launched/published	Publication year of included documents	Policy type	Primary issuing authority	Further information
Mental health	National/District Mental Health Programme (N/DMHP) [68, 115]	1982	2015/2017	Programme	Ministry of Health and Family Welfare	The programme has been subsumed under the NHM
	National Mental Health Policy [7, 116]	2014	2014	Strategic vision	Ministry of Health and Family Welfare	
	Mental Healthcare Act (MHCA) [57, 63, 117]	2017	2017/2018	Legislation	Ministry of Law and Justice	
Old age	Integrated Programme for Older Persons (IPOP) [118]	1992	2016	Programme	Ministry of Social Justice and Empowerment	The programme’s latest revision of 2016 was included
	The Maintenance and Welfare of Parents and Senior Citizens Act (MWPSCA) [64]	2007	2007	Legislation	Ministry of Law and Justice	
	National Policy for Senior Citizens (NPSC) [54, 56]	2011	2011/2014	Strategic vision	Ministry of Social Justice and Empowerment	The NPSC 2011 is in a draft stage
	National Programme for the Health Care of the Elderly (NPHCE) [66, 69, 119]	2011	2011/2016	Programme	Ministry of Health and Family Welfare	The NPHCE has been subsumed under the NHM
General health	National Health Mission (NHM) [36, 38, 65, 120–124]	2005	2009/2010/2012/2013/2015	Strategic vision and guidelines	Ministry of Health and Family Welfare	The included documents primarily provide information about the NRHM, the NUHM, and CHWs
	National Programme for Control and Prevention of Cancer, Diabetes, Cardiovascular Diseases and Stroke (NPCDCS) [125–127]	2010	2010/2016/2017	Programme	Ministry of Health and Family Welfare	The NPCDCS has been subsumed under the NHM
	Indian Public Health Standards (IPHS) –Guidelines for PHCCs and SHCs [40, 128]	2012	2012	Guidelines	Ministry of Health and Family Welfare	The IPHS are a health system strengthening component of the NHM
	National Action Plan and Monitoring Framework for Prevention and Control of Noncommunicable Diseases (NCDs) [129]	2013	2013	Guidelines	Ministry of Health and Family Welfare	The Framework is complimentary to the NPCDCS
	National AYUSH Mission (NAM) [70, 130]	2014	2014	Strategic vision	Ministry of AYUSH	The Department of AYUSH in the MoH&FW, which oversaw the NAM, became the Ministry of AYUSH in 2014
	Comprehensive Primary Health Care (CPHC) [61, 67, 131]	2015	2015/2018	Strategic vision and guidelines	Ministry of Health and Family Welfare	
	Rights of Persons with Disabilities Act (RPDA) [132–135]	2016	2016/2017/2018	Legislation	Ministry of Law and Justice	
	National Health Policy (NHP) [62, 136]	2017	2017	Strategic vision	Ministry of Health and Family Welfare	

The table was developed based on Table 2 in Kafczyk and Hämel [6]

*CHWs* Community Health Workers, *CPHC* Comprehensive Primary Health Care, *NHM* National Health Mission, *NRHM* National Rural Health Mission, *NUHM* National Urban Health Mission, *PHCC* Primary Health Care Centre, *SHC* Sub Health Centre

To analyse strategies and approaches at the primary care level in more detail, we conducted the study presented here.

Public health policies are acknowledged in this study as instruments that outline a vision and path for developing older people’s access to mental healthcare and reducing the burden of mental illness [48, 49]. In particular, the organization of healthcare is the result of and is regulated by health policy decisions [50]. Policy translates into social reality, and social reality shapes policy [51]. It should be noted, however, that policies themselves shape an ideal picture of reality – the implementation of these policies depends on an abundance of factors that are not objects of this study.

### Search strategy and inclusion of policy documents

The search strategy and inclusion of policy documents have already been presented in the methods section of our previous study [6]. For this second in-depth analysis, however, for reasons of clarity and comprehensibility, relevant steps and differences are (again) summarized here. A web search of federal-level ministries’ websites and the WHO’s MiNDbank database was conducted between February 2019 and February 2020 without year restrictions; it aimed to identify key strategic public policy documents in the three policy fields (see also [6]). In addition, as an important validation step, the first author consulted key informants (researchers, practitioners, and policy makers) in mental health and geriatric care in India to determine what relevant national-level policy documents for mental healthcare for older people exist. Identified policy documents were screened based on the following selection criteria (see also [6]):

- English language*:* federal-level documents are usually available in English. Hence, only documents in English were included.
- Policy documents with national-level implications: only documents from the federal government were included, as it is the government’s role to provide a strategic direction and guidance through policies [52].
- Relevant to old age mental healthcare*:* important in this step was that the absence of a clear intention or action or simply failure to address the issue can also be regarded as policy [6, 53]; i.e., documents relevant to the architecture of primary mental healthcare for older people that lack concretization of this issue were also included.
- Approved by concerned authorities and in force*:* two exemptions from this rule are (a) the National Policy for Senior Citizens 2011 [54] and (b) the Notice Draft Rules and Regulations under the Mental Healthcare Act (MHCA) 2017 [57] (for further details see [6]). These policies’ statuses were considered in the data interpretation.

In contrast to our first study [6], we excluded two documents for this in-depth analysis: the National Policy on Older Persons (1999) and the National Policy for Persons with Disabilities (2006). These two documents were not included because they do not inform the research questions of this study.

In total, of 70 screened policy documents, 39 were included in this study (see Table 2). The documents were published between 2007 and 2018; older documents were not included because they were not pertinent to the presently envisioned design of the primary mental healthcare system. Each document defines and lays out a vision and/or objective for the mental health, old age, and general healthcare of the population and/or a corresponding framework to reach the outlined vision/objectives; included are laws, strategic plans, and action programs.

### Data extraction and analysis

A thematic analysis was conducted [58, 59]. With reference to the research questions, four categories were deductively determined: (1) the role of PHC, (2) the organization of mental healthcare in PHC, (3) approaches to and strategies for mental health services for older persons, and (4) the role of family caregivers. For each category, relevant passages in the documents were coded, extracted, and organized in data collection sheets for in-depth interpretation.

In the first step, relevant passages were interpreted and summarized in their respective policy fields to identify the most salient themes. In a second step, the results were triangulated at a meta-level by examination of the similarities or differences between policy fields. In a third step, categories and corresponding themes were described, drafted, and further refined in discussions held by the research team. As a result, six categories or themes were extracted and are presented in the results section. This method is in line with the READ approach to document analysis devised by Dalglish, Khalid [60], which stands for (a) ready your material, (b) extract the data, (c) analyse the data, and (d) distil the findings.

## Results

### A conflicted vision for a community-based and comprehensive primary healthcare-based approach to old age mental healthcare

Primary mental healthcare is a topic that is given increasing attention over time in the analysed policies, but it is emphasized less in old age policies than in mental health and general health policies. Newer policies – starting in 2015 – outline its advantages in a wider vision of a PHC-oriented health system that should allow for improved prevention and cost-effectiveness with a reduced burden on secondary and tertiary care (ex. [7, 61, 62]).

As a milestone, the MHCA 2017 [63] mandates the integration of mental health at all levels, *including* PHC, into all public health programs. The Act also stresses the benefits of strong primary mental healthcare; however, it remains vague about the role and contributions of PHC in the Indian mental health system (for further details see [6]). This holds true for the Maintenance and Welfare of Parents and Senior Citizens Act (MWPSCA) from 2007 [64]. Both acts are more specific about mental healthcare at the secondary and tertiary levels. In contrast, the National Health Policy 2017 [62] delineates a much clearer picture by suggesting a shared responsibility across the care levels. At the PHC level, screening for mental illness, detection of cases, and referral to specialists as well as the provision of continuous care, including follow-up of mentally ill patients, should be conducted. The secondary and tertiary levels should concentrate on the diagnosis and treatment of mental illnesses [61, 65]. However, a general concern across policy fields is the insufficient capacity and insufficient funding of PHC, as stated in the National Programme for the Health Care of the Elderly (NPHCE): “At the primary care level, the infrastructure is grossly deficient” ([66], p. 3). This suggests that the expansion of primary mental healthcare for older people may be difficult to realize.

In all policy fields, the envisioned PHC is ‘community-based’ and ‘comprehensive’ (ex. [7, 62, 66]). The NPHCE ([66], p. 4) states the need “[t]o provide an easy access to promotional, preventive, curative and rehabilitative services to the elderly through community based primary health care approach”. The MHCA 2017 strengthens community-based care through the right of persons with a mental illness – including older persons – to live in the community and with family. Consequently, providing access to mental health services close to peoples’ homes is an obligation of Indian states to:

“[…] ensure that no person with mental illness (including children and older persons) shall be required to travel long distances to access mental health services and such services shall be available close to a place where a person with mental illness resides” ([64], p. 10).

A shift from selective to more comprehensive PHC is demanded more emphatically over time. This, however, appears difficult to accomplish, since “[t]he primary focus of Sub-centre [at the PHC level] remains the Reproductive and Child Health (RCH) services” as the Indian Public Health Standards (PHS) state ([40], p. vii), putting inclusive care for older persons in a tenuous position. Consequently, the integration of specialists to address older people’s needs at the PHC level as well is seen as beneficial. The NPHCE ([66], p. 28, 3) states,

“[i]n view of their [older persons, the authors] rapidly increasing number with varied health, economic and psycho-social needs. […] Their health problems […] need specialist care from various disciplines e.g. ophthalmology, orthopedics, **psychiatry**, cardiovascular, dental, urology to name a few. Thus a model of care providing comprehensive health services to elderly at **all levels** of health care delivery is imperative […].” (bold added by the authors for emphasis).

The new care model of Health and Wellness Centres (HWCs) is promoted, e.g., in the National Health Policy (NHP) 2017 [62] as the main mode for the delivery of comprehensive PHC to best meet the complex care needs of the population at the community level. The HWCs complement existing PHC structures and partially replace them [61]. Care at HWCs should be delivered “[…] in ways that take into cognizance the dignity of the individual, the needs and circumstances of the family, and the culture of the community” (Ayushman Bharat – Operational Guidelines for Comprehensive PHC [67], p. 41). However, whether and how HWCs are to offer mental healthcare, particularly for older persons, are not specifically discussed in the policies.

### Promotion of mental healthcare through the community

Self-organized care and community participation in healthcare planning, implementation, monitoring, and evaluation are integral to the understanding of community-based care in the policy documents. In particular, mental health policies emphasize that mental healthcare is to be shaped by the community itself. Local governments are expected to collaborate with persons with mental illness and their families, both in the planning and the execution of service delivery. The NHP 2017 ([62], p. 20) envisages “[…] a sustainable network for community/locality towards mental health.” Old age polices are not specific to mental health in this regard. The National Urban Health Mission (NUHM) speaks expressly of a “communitization process” ([38], p. 5) to be realized by the Village Health Sanitation and Nutrition Committees (VHSNC) as a key institution for community health governance.

“In order to build community support and offer good healthcare to the vulnerable sections of the society like the marginalised, the socially excluded, the poor, the old and the disabled, the policy recommends strengthening the VHSNCs and its equivalent in the urban areas” (NHP 2017 ([62], p. 7)).

In addition, documents from all policy fields highlight the promotion of self-help groups in communities as essential for community-based PHC. However, documents that refer specifically to Self Health Care Groups of Elderly Persons (e.g., IPHS for SHCs [40]) fail to outline their possible contribution to mental health.

### Strengthening community health workers as a resource for old age mental healthcare

Outreach activities to promote health and prevent disease in vulnerable populations are a frequently discussed measure, especially in health and old age policies. Outreach is commonly understood as the community-based work of CHWs. The documents envisage an expansion of the scope of CHWs’ practice beyond the traditional focus on family and child health and communicable disease care to addressing the care needs of older persons; the monitoring of the health of older people includes the detection of their mental health needs [36, 67]. For example, CHWs are assigned a special role in the NPHCE’s approach to “[…] provide elderly persons or the family […] [with] information on interventions such as: Health Education related to healthy ageing, environmental modifications, nutritional requirements, life styles and behavioural changes*”* ([66], p. 11). Among CHWs, ASHAs in particular – supported by other health workers [62] – are envisioned as supporting persons and families affected by mental health problems. However, there are also critical observations that ASHAs’ “[…] role continues to be circumscribed towards promoting utilization of a limited set of RCH [Reproductive Child Health, the authors] programmes, representing a missed opportunity for the ASHA to play a key role in the primary health care team” (Task Force on Comprehensive PHC Rollout [61], p. 21)*.* Consequently, the “[r]ole of ASHA at village level need to be worked out particularly for mobilize of the elderly to attend camps and home-based care for bed-ridden elderly” (NPHCE ([66], p. 11) so that their new expected role is not undermined.

The provision of home-based care is a common task ascribed to CHWs in old age policies. The NPHCE envisages home care as a central means to provide access to care for home-bound and bedridden older persons, those who have disabilities and those who have post-inpatient care needs, including needs related to rehabilitation and recovery. A core strategy to achieve the program’s objectives is a “[c]ommunity based primary health care approach including domiciliary visits by trained health care workers” (NPHCE ([66], p. 5).

### Supported family-led care for (older) people with mental health problems

The policy documents unanimously recognize families as the primary source of caregiving for older people that enables them to live as long as possible in their accustomed environment (ex. [54]). “Provision of care by primary care team will be based on principles of family led care […]” (Ayushman Bharat Operational Guidelines for Comprehensive Primary Health Care ([67], p. 13). This must be seen in light of the obligation of families to provide care for (older) family members as established by the MWPSCA 2007 and reinforced in the MHCA 2017, which declares:

“Where it is not possible for a mentally ill person to live with his family or relatives, or where a mentally ill person has been abandoned by his family or relatives, the appropriate Government shall provide support as appropriate including legal aid and to facilitate exercising his right to family home and living in the family home” ([63], chapter V, 19(2), p. 11).

Within the mental health field, the National Mental Health Policy 2014 suggests a family-centric care model that includes support to help family caregivers maintain their functioning; in this way, it recognizes the heavy burden that care for persons with mental health problems places on families. In line with this, the DMHP ([68], p. 8) envisages that “[f]amily members must also be involved in psychosocial interventions as much as possible.”

Different means of support for families are envisaged. The MHCA 2017 ([63], chapter V, 18(3), p. 9) obliges state governments to support the families of persons with mental illness, which shall include “provision for mental health services to support family of person with mental illness or home based rehabilitation.” Another means of support for families caring for older relatives, especially in mental health and old age policies, is indirect financial transfers to family caregivers (i.e., tax benefits). The National Mental Health Policy 2014 ([7], p. 16) states “[t]here is a need to implement programmes to address the economic needs of this very important stakeholder group.”

Additionally, across all policy fields, a dominant approach to encouraging and supporting the family is improving the capacity of family caregivers through information and training: “Support family in identifying behavioural changes in elderly and providing care” (Ayushman Bharat Operational Guidelines 2018 ([67], p. 19). However, it is notable that support for families caring for older relatives is not specifically discussed in the context of mental health in the field of old age policy.

As outlined above, policies see professional care as supplementing and supporting family care and as enabling the family to fulfil its overall function. Policies only loosely touch on support for older persons who lack informal social support. The National Mental Health Policy 2014 [7] recommends domiciliary care for persons with mental health problems who lack family support to facilitate recovery.

### Ayurveda, Yoga and Naturopathy, Unani, Siddha, Sowa-Rigpa and Homeopathy are of increasing importance in mental healthcare

Over the period examined, AYUSH gained increasing importance in policies as a beneficial resource for (primary) mental healthcare. Since about 2014, AYUSH has been a prominent topic across policy fields. For example, the National Mental Health Policy 2014 ([7], p. 14) mentions that “[p]ractitioners of Ayurveda and Yoga systems are a resource who need to be included as activists for promotion of mental health.” Similarly, the NPHCE 2011 mentions plans for the “[d]evelopment of a service for 'yoga' therapy for senior citizens especially for 75 + population in National Centers for Ageing and Regional Geriatric Centres […] [and the coordination, the authors] […] with local AYUSH practitioners […]” (Continuation and Expansion of Tertiary Care Level Activities of NPHCE [69], p. 2 – 3). In the MHCA 2017, AYUSH practitioners and facilities are included in definitions of mental health professionals and mental health establishments, solidifying their elevated status within the rights-based framework for mental healthcare. The Report of the Task Force on Comprehensive PHC [61] explains that AYUSH providers are also intended to be elevated to mid-level health providers at the PHC level. Other non-AYUSH care providers, including CHWs and mid-level providers, are expected to receive capacity building in AYUSH practices.

Similarly, the National AYUSH Mission (NAM) from 2014 [70] proposes the co-location of AYUSH services in PHC setups and proposes that AYUSH staff support other health programs. Interestingly, however, the NAM itself is not specific to mental health or old age, indicating a lack of intersectoral collaboration and a shared vision in policymaking.

### Human resource development as a major topic in primary mental healthcare

As a part of a broader policy discussion on strengthening the capacity of the PHC system, capacity building for health professionals and CHWs in mental health and geriatric care is a common theme in the analysed policy documents.

Mental health policies point to the gap between the need for and availability of trained mental health professionals. The MHCA 2017 [63] stands out in this regard, as it introduced an obligation for governments to address human resource shortages in mental healthcare in terms of both quantity and quality. The DMHP [68] envisions a manpower development scheme to provide basic mental healthcare at the PHC level. The National Mental Health Policy 2014 [7] adds that training in the biomedical approach and the psychosocial approach to care are equally important to providing better care for patients and caregivers.

In health policies, a ‘diversification of skills approach’ is pursued: “Human resources posted at all levels would be trained to be multi-skilled. Mid level providers would be trained and certified in a Bridge Course designed to ensure public health and primary health care competencies” (Task Force on CPHC Rollout 2015 [61], p. 3). One important component of training is the “[…] understanding of marginalization and the need for social mobilization to address the most vulnerable” (Task Force on CPHC Rollout 2015 [61], p. 8) to ensure equitable access to care. However, educating health workers in old age mental healthcare is not thematized. Similarly, while the NPHCE [66] has included a training component in healthcare for older persons at the PHC level – for doctors, nurses and CHWs – mental health is absent from these plans, which are to be prepared and implemented through the National Programme for Prevention and Control of Cancer, Diabetes, Cardiovascular Diseases and Stroke (NPCDCS). This could undermine the strengthening of old age mental healthcare at the PHC level.

## Discussion

In this policy analysis study, we explored the emerging trend of primary mental healthcare for older persons in India. Of interest was the assigned role of the PHC system in general as well as concrete strategies for and approaches to primary mental healthcare for older persons in particular. We also paid special attention to the role of family caregivers in our analysis.

### Strengthening the role of primary mental healthcare for older persons through traditional and existing resources

Our analysis provides evidence that comprehensive community-based primary mental healthcare – focusing vulnerable population groups including older persons – was strengthened significantly in the period 2007–2019, as corroborated by Patel, Xiao [20]. Moreover, the envisioned approaches and strategies are in line with the tradition of community-based approaches to mental healthcare in India [1] and a healthcare system built around the needs of vulnerable populations such as older people [33].

The envisaged role of mental healthcare for older people in PHC focuses on the prevention and detection of mental health issues and is supported by follow-ups to enable continuous care in the community. It is strengthened through (a) integrating CHWs into primary mental healthcare, (b) empowering the community to participate in healthcare planning, implementation, and monitoring, (c) supporting the family in a family-led approach to mental healthcare, and (d) integrating traditional AYUSH services into primary mental healthcare.

### Involving community health workers in old age mental healthcare

A ‘task sharing’ approach is proposed in the analysed policies that transfers mental healthcare tasks from health professionals to CHWs. Accordingly, CHWs’ scope of work will expand to addressing older people’s health and involvement in mental healthcare. Older persons and their families are especially expected to benefit from home visits to identify older persons vulnerable to mental health issues and to support family caregiving. CHWs are well established in India; they have a strong presence in communities and often come from the communities they serve and thus know where older persons live. This strategy could, therefore, help reach older persons, mitigating the large mental health treatment gap for older persons, especially in rural areas. However, the analysed policy documents do not provide robust guidance on how CHWs can be enabled and supported to provide old age mental healthcare in India, indicating a gap between the policies’ vision and the concrete steps needed to implement the vision. Mental health interventions by CHWs and other lay health workers have shown promising results in India [71] and other LMICs [72–74] if personnel receive sufficient training and supervision [72, 75], including the early identification of persons with mental health problems and case management [14, 76]. Policies are needed that elaborate on how health workers should be trained in old age mental healthcare.

CHWs could also strengthen the link between the community and the mental healthcare system by overseeing and supporting community action for mental healthcare, as proposed in the policies. These workers offer the opportunity not only to provide instrumental support but also to strengthen community participation, an approach that is gaining attention in other LMICs such as Brazil [77], but also increasingly in high-income countries, given the challenges facing aging societies in healthcare [78]. However, it is important to not overburden CHWs with too many tasks. CHWs already have a high workload with health priority tasks [74, 79], and tend to focus on maternal and child health and family planning [35, 40]. It must be questioned whether this is a good starting point to further develop and expand CHWs’ contributions to old age mental healthcare. Moreover, a more context-specific evaluation might be fruitful as policies drawing on CHWs as a resource of the PHC-system have focused on the conditions in rural India; with the adaptation of such approaches for urban and metropolitan regions [38], the different contexts of these communities should be considered by policy planners and health practitioners.

The role of CHWs in old age mental healthcare requires more attention by policymakers to prevent a missed opportunity. Opportunities for and obstacles to the involvement of CHWs in old age mental healthcare should be investigated to enable a clearer formulation of what works and what does not.

### The challenging path of coproduction between formal and informal care

Across the policy fields, a familialistic policy approach prescribing and, to some extent, supporting a high degree of family responsibility in caring for older persons is not contested. The family-centred care and support vision for older people is rooted in the Indian tradition that families care for older family members [23]. Its appropriateness must be reconsidered in light of the parallel existence of traditional family-based care systems and the growth of ‘modern’ nuclear family systems in which increasing numbers of older persons receive little informal support [13, 14, 25, 80]. This change in family norms and abilities is, however, not clearly addressed in policies. Policies need to better account for the new social reality. One group in need of particular attention is older widows the most marginalized and often ostracized members of Indian society [81]. Though older widows are identified as a group vulnerable to mental health issues in the analysed policies, the documents fail to outline plans detailing how mental health support in the absence of family care can be shaped. This should be a priority in future health planning.

A general concern with respect to the adequacy of a family-led approach to old age mental healthcare is that families do not prioritize the mental health of older people. Families might be discouraged from seeking support outside of the family and may not think of seeking professional care [14, 27, 82]. Ill health, including mental illness, in old age in India is often disregarded and misunderstood as a normal part of ageing by families and older persons themselves [14, 15, 83, 84], with home remedies and self-medication taking precedence over professional help [15]. Studies have shown that families and older persons seek to protect their reputation because of the (social) stigma attached to mental health issues [14]. Stigma is one of the main barriers to seeking treatment for mental health problems [85]. Policies should acknowledge stigma as a major barrier to old age mental healthcare, even in the family context, and lay out plans to address it. Community-level interventions are a common strategy in India to reduce stigma [86], and this level should be considered important.

### AYUSH: an opportunity for age- and culturally sensitive primary mental healthcare?

Interestingly, although the public health service model in India is based on Western models of care [87], there is a strong and increasing tendency in policies to integrate traditional AYUSH services into primary care. The integration of AYUSH represents an opportunity to shift to a more holistic understanding of mental healthcare and has been described as a cost-effective strategy [88]. The strategy of the integration of AYUSH into PHC and mental healthcare intersects, again, with established practices, given that much of the mental healthcare burden for older people is already falling on traditional care structures [21, 24, 89]. In addition, there is evidence that traditional and spiritual practices have a beneficial impact on older people’s health and well-being, which might be connected to the deeply religious and collectivist nature of Indian culture [90, 91]. For example, Sivaramakrishnan, Fitzsimons [91] have shown in their systematic review and meta-analysis that yoga practice has a beneficial effect on older people’s mental health. It has also been argued that the integration of traditional and complementary medicine approaches offers an opportunity for a more health-promoting model of PHC [92].

Moreover, incorporating already frequented care structures is an opportunity for a more comprehensive approach to primary mental healthcare for older people and can be seen as an opportunity to promote universal health coverage [93]. However, AYUSH policies themselves must be much more specific to the mental health of older people for their potential to be realized. In addition, the integration of AYUSH into PHC must always be considered against the background of the needs and preferences of the communities and the regional and cultural diversity of the population in India. Albert and Porter thus criticize a ‘forced pluralism’ and ‘top-down approach’ ([91, 94], p. 5, p. 7) of Indian AYUSH policies; this approach could negatively impact acceptance of AYUSH among users but also other healthcare providers. As PHC policies in India themselves emphasize, community participation in healthcare planning and implementation is integral to achieving community-based care. It is also important to encourage and support cooperation between conventional healthcare professionals and AYUSH practitioners [93] to avoid a fragmentation of care.

### Towards an unambiguous and realistic vision of old age primary mental healthcare

While the policies indicate that mental healthcare for older people will be strengthened in PHC, they are vague in terms of outlining a clear and realistic way forward. One major challenge is the interplay between the PHC system and higher levels of care. Despite the policies’ emphasis on PHC in responding to the mental health needs of older persons, the secondary and tertiary care levels are still much more strongly contoured. The diagnosis and treatment of mental illnesses are foreseen almost exclusively in secondary and tertiary care settings. In rural areas, where most older people live [18], PHC facilities are usually the only ones within reach and (geographically) accessible. Ideally, mental health problems in LMICs are addressed at the PHC level [95, 96]. This problem is compounded by health financing in India, which is skewed towards secondary and tertiary care [20, 97]. For example, the government-sponsored national 'flagship' scheme Pradhan Mantri Jan Arogya Yojana (PM-JAY), which provides health insurance coverage primarily to vulnerable population groups, is limited to secondary and tertiary care [98]. This scheme should be extended to cover PHC settings as well, as envisaged by the MHCA 2017 [99]. Generally, the interplay between the PHC and other levels of care needs to be worked out in policies more clearly to reflect the reality on the ground.

Furthermore, policies themselves express concern that the primary (mental) healthcare system in India is functioning poorly, particularly in addressing the needs of older persons [14]. To successfully integrate mental healthcare into PHC, more resources and capacities at the PHC level are required in urban as well as rural areas across India. Realizing the policies’ vision requires adequate public financing for PHC and mental healthcare as an integral part of a comprehensive PHC. Otherwise, the large disparity between the policies’ vision and reality will hinder implementation. The reluctance to increase public health spending creates doubt that a properly functioning public mental healthcare system can be established. However, there is optimism that new legislative requirements outlined in the MHCA 2017 can be used to channel funding towards mental health [100].

### Policy recommendations

- It will be important to develop an intersectoral and collaborative policy field for old age mental healthcare at the PHC level.
- In view of changing social norms and the breakdown of traditional family care structures, it will be important to develop clear support strategies for the growing group of older persons with mental healthcare needs who have little informal support while also supporting families that care for the mental health of older family members.
- Strengthening PHC in India will be crucial to enable the integration of mental healthcare as a part of comprehensive PHC.
- It will be important to create congruent politics and unambiguous implementation guidelines suited to state and local contexts to transform policy into practice considering well-known implementation barriers such as political and bureaucratic commitment [101], which may vary from state to state, and the stigma attached to mental issues, particularly in older age.

### Limitations

It is important to bear in mind that the Indian constitution regards health as a state matter. The policy documents considered in this study provide guidance on a national level, but the policies’ interpretation and implementation are subject to state processes and depend on cooperative federalism. With regard to legislative acts, however, all states are bound to secure the rights stated within.

The implementation of policies was not the focus here, but it is known that policies are usually not adopted to the letter and that one of the main challenges is the transfer of evidence into practice [102, 103]. The body of literature from India on the implementation of the public health programs discussed in this paper, such as the N/DMHP [19, 20, 28, 43, 104, 105], points to the struggle to achieve these programs’ targets. While evidence on how to implement mental health practices effectively is increasing in LMICs [106] and India, more research is needed to determine how to move from ideal concepts to real change and what the actual uptake of policies and programs on a state and district level is, considering the heterogeneous influencing factors at play.

It was the intention of this study to point out strengths and weaknesses in the policies’ formulation, which often brings about implementation ambiguities. Since nongovernmental and private sector policies were not included, because these sectors do not have the legal mandate to develop policies for the entire population [53], it was not possible to generalize to the public and private healthcare system as a whole. Nevertheless, private actors were involved in the drafting of some of the included policy documents, allowing us to infer at least to a certain degree that the formulations are also valid for the private sector.

### Conclusion

This study shows the strengths and weaknesses of the strategies and approaches outlined in public health policies for primary mental healthcare for older persons in India. Overall, India’s approach to mental healthcare for older persons is based on family- and community-based care, integrating traditional care structures into a new vision of comprehensive PHC. To realize this vision, strengthening PHC is essential. While steps have been taken that are important to strengthening this sector in India and that could be inspiring for other LMICs that face similar challenges, mental healthcare for older persons in India is still in its infancy. Unambiguous and integrated policy approaches are needed to address the mental healthcare needs of older persons. The mental health of older persons and the role of the family, as an important resource in mental healthcare, must not be neglected in care policies to build a socially just and equitable health system. Further research should empirically focus on the opportunities and challenges that older people face in terms of the current policy approaches.

## Data Availability

Data sharing is not applicable to this article, as publicly accessible policy documents were analysed.

## Abbreviations

ANM: Auxiliary Nurse-Midwife
ASHA: Accredited Social Health Activist
AWW: Anganwadi Worker
AYUSH: Ayurveda, Yoga and Naturopathy, Unani, Siddha, Sowa-Rigpa and Homeopathy
CHC: Community Health Centre
CHW: Community Health Worker
CPHC: Comprehensive Primary Health Care
DMHP: District Mental Health Programme
HWC: Health and Wellness Centre
LMIC: Low- and Middle-Income Country
MHCA: Mental Healthcare Act
MoH&FW: Ministry of Health and Family Welfare
MWPSCA: The Maintenance and Welfare of Parents and Senior Citizens Act
NAM: National AYUSH Mission
NCD: Noncommunicable Disease
NHM: National Health Mission
NHP: National Health Policy
NMHP: National Mental Health Programme
NPCDCS: National Programme for Prevention and Control of Cancer, Diabetes, Cardiovascular Diseases and Stroke
NPHCE: National Programme for the Healthcare of the Elderly
NRHM: National Rural Health Mission
NUHM: National Urban Health Mission
PHC: Primary Healthcare
PHCC: Primary Healthcare Centre
PHS: Public Health Standards
SHC: Sub-Health Centre
VHSNC: Village Health Sanitation Nutrition Committee
WHO: World Health Organization
